# NKX2-5 Regulates the Expression of β-Catenin and GATA4 in Ventricular Myocytes

**DOI:** 10.1371/journal.pone.0005698

**Published:** 2009-05-28

**Authors:** Ali M. Riazi, Jun K. Takeuchi, Lisa K. Hornberger, Syed Hassan Zaidi, Fariba Amini, John Coles, Benoit G. Bruneau, Glen S. Van Arsdell

**Affiliations:** 1 Labatt Family Heart Centre, Hospital for Sick Children, Toronto, Ontario, Canada; 2 Fetal Cardiovascular Program, Pediatric Heart Center and Fetal Treatment Center, School of Medicine, University of California San Francisco, San Francisco, California, United States of America; 3 Division of Cardiology, University Health Network, University of Toronto, Toronto, Ontario, Canada; 4 Gladstone Institute of Cardiovascular Disease, Department of Pediatrics, University of California San Francisco, San Francisco, California, United States of America; 5 Division of Cardiovascular Research, Global-Edge Institute, Tokyo Institute of Technology, Yokohama, Japan; National Institute on Aging, United States of America

## Abstract

**Background:**

The molecular pathway that controls cardiogenesis is temporally and spatially regulated by master transcriptional regulators such as NKX2-5, Isl1, MEF2C, GATA4, and β-catenin. The interplay between these factors and their downstream targets are not completely understood. Here, we studied regulation of β-catenin and GATA4 by NKX2-5 in human fetal cardiac myocytes.

**Methodology/Principal Findings:**

Using antisense inhibition we disrupted the expression of NKX2-5 and studied changes in expression of cardiac-associated genes. Down-regulation of NKX2-5 resulted in increased β-catenin while GATA4 was decreased. We demonstrated that this regulation was conferred by binding of NKX2-5 to specific elements (NKEs) in the promoter region of the *β-catenin* and *GATA4* genes. Using promoter-luciferase reporter assay combined with mutational analysis of the NKEs we demonstrated that the identified NKX2-5 binding sites were essential for the suppression of β-catenin, and upregulation of GATA4 by NKX2-5.

**Conclusions:**

This study suggests that NKX2-5 modulates the β-catenin and GATA4 transcriptional activities in developing human cardiac myocytes.

## Introduction

During early development myocardial cells within primary and secondary heart fields form under influence of bone morphogenetic proteins (BMPs), fibroblast growth factors (FGFs), and proteins from Wnt family, that initiate expression of a cascade of cardiac-associated transcription factors including NKX2-5, Isl1, MEF2C, GATA4, and β-catenin and their downstream targets (reviewed in [1], [2]). The cardiac function of these transcription factors and their regulation is only partially understood. The importance of Nkx2-5, GATA4, and MEF2C in cardiac development has been demonstrated in many studies [3], [4], reviewed in [5]; the role of Wnt/β-catenin pathway in cardiogenesis has recently begun to be unraveled [6]. Although the early studies were pointing at an inhibitory role for β-catenin dependent Wnt pathway on cardiogenesis [2], [7]–[9], more recent studies have shown a biphasic role where β-catenin is necessary at earlier stages of cardiomyogenesis and inhibitory at later stages of heart development. Furthermore, cardiac-specific deletion of β-catenin has proved to be deleterious when β-catenin is deleted in cardiac cells originated from the secondary heart fields [10], suggesting spatial difference in gene cascades that control cardiac myocyogenesis. Since NKX2-5 transcription factor is one of the earliest genes expressed in the heart cells we hypothesized that β-catenin might be regulated by NKX2-5 in cardiac myocytes. Analysis of promoter regions identified candidate NKX2-5 binding elements (NKEs) in *β-catenin* and *GATA4* genes. To test if β-catenin and GATA4 are regulated by NKX2-5, endogenous NKX2-5 expression was knocked down by expressing antisense NKX2-5 RNA (XKN) in human fetal ventricular myocytes. This study shows that β-catenin and GATA4 transcription factors are regulated by NKE sequences in the promoter region of these genes. In addition, we confirm direct physical interactions between NKX2-5 and NKEs in the promoters of β-catenin and GATA4 as demonstrated by electrophoretic mobility shift, chromatin immunoprecipitation, and luciferase promoter assays. This study supports the essential role of NKX2-5 in maintaining the cardiac gene expression program and suggests direct regulation of β-catenin and GATA4 by NKX2-5 in human cardiomyocytes.

## Results

Identification of NKX2-5 binding sites in the promoters of β-catenin and GATA4 genes

**Figure 1:**
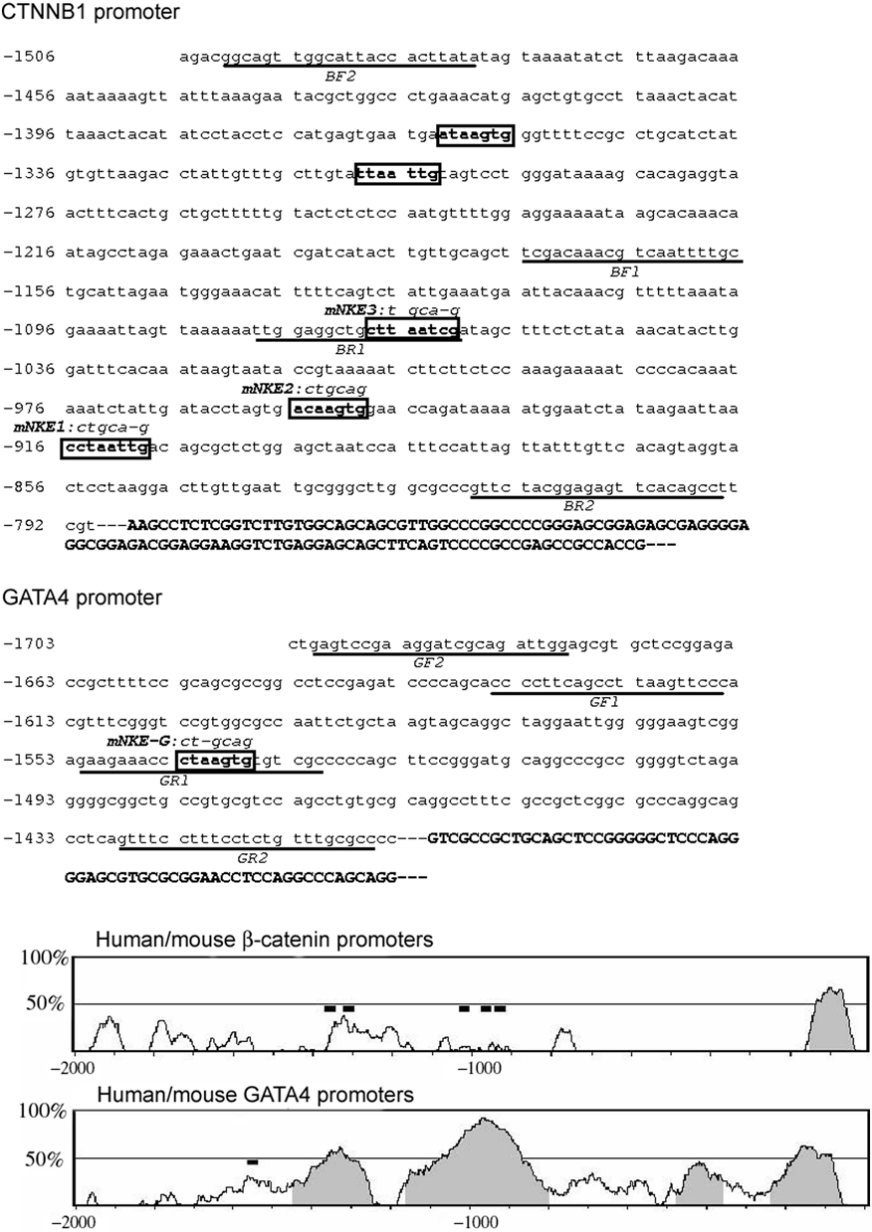
Identification of NKX2-5 binding sites in β-catenin (CTNNB1) and GATA4 promoters. The promoter sequence of *β-catenin* and *GATA4* genes contains candidate NKX2.5 binding sites (boxed sequences). The first exons of *β-catenin* and *GATA4* are indicated with capital-bold letters and primers: BF1, BR1, GF1, and GR1 used in ChIP analysis are underlined. Primers GF2, GR2, BF2, and BR2 (underlined) delineate the region cloned and used in luciferase assay. The base changes in the NKE sequences (mNKEs), used in gene reporter assays have been shown. The bottom panel shows the level of DNA sequence conservation between human and mouse 2-kb upstream of β-catenin and GATA4 first exons. Shaded areas demonstrate very high level of conservation. Black boxes indicate the identified NKEs.

The genomic sequence surrounding the first exons of human *β-catenin* and *GATA4* genes were searched for NKX2-5-binding consensus sequence (NKE), TNAAGTG [11], using TFSEARCH. The 2 kb sequence immediately upstream of the first exon of human *β-catenin* gene (CTNNB1) [12] was searched for candidate NKEs. Analysis of this sequence revealed candidate NKEs in positions −900 to −1400 (Fig. 1). Binding sites for USF (upstream stimulating factor) and other transcription factors such as SP-1, P300, ADR1, MyoD, and GATA1 were also found in this region (not shown). Similar analysis on the sequence surrounding the first exon of *GATA4* gene was performed and a candidate NKEs in position -1540 was identified. The identified NKEs are located in the regions partially conserved between human and mouse when the promoter sequences are aligned using rVISTA (Fig. 1).

### NKX2-5 regulates the expression of β-catenin and GATA4 in cardiac myocytes

**Figure 2:**
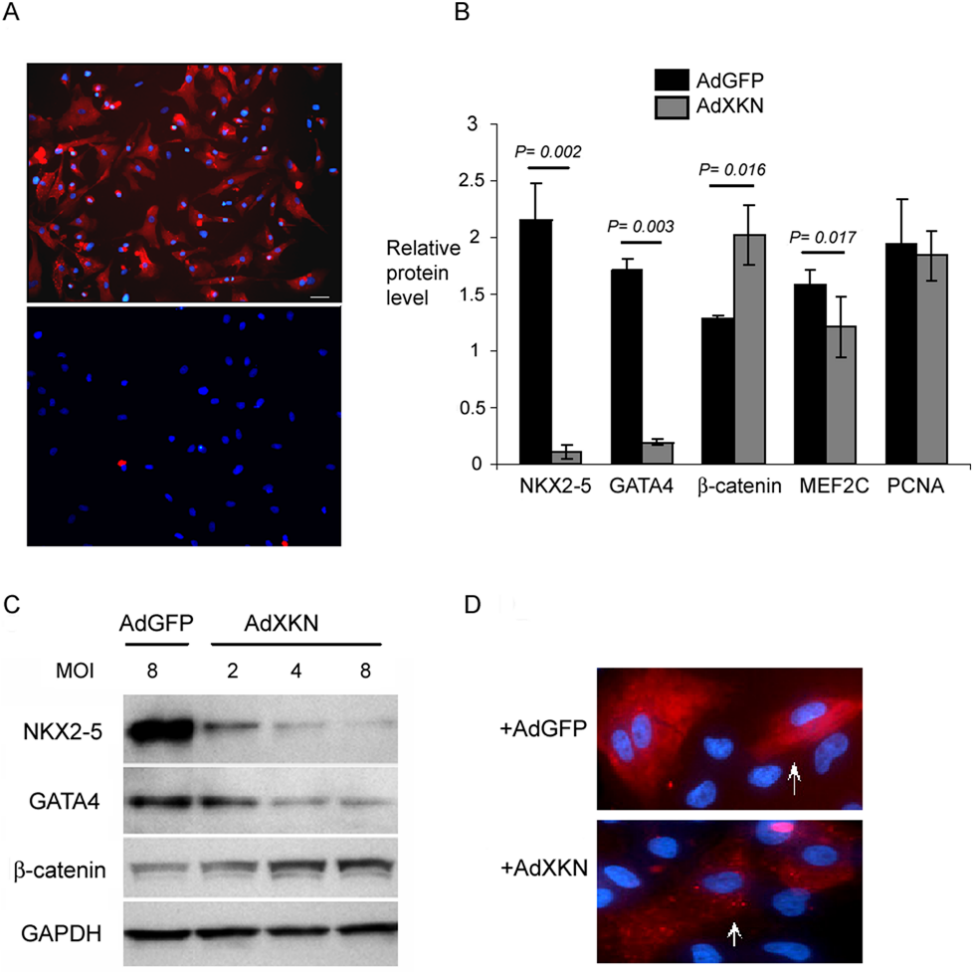
Down-regulation of NKX2-5 alters expression of cardiac-associated genes. (A) Cardiac myocytes and fibroblasts isolated from human fetus at 20-22 weeks of age and cultured for 5 days were immunostained with anti-MyHC antibody (MF-20) to determine the percentage of myocytes in the preparations. The isolated myocyte culture contained >90% MyHC^+^ cells while the fibroblasts revealed <5% MyHC^+^ cells. (B) The graph summarizes the western blot analysis after treatment with AdGFP and AdXKN (antisense Nkx2-*5* RNA) at 8 MOI, and immunoblotting with NKX2-5, GATA4, β-catenin, MEF2C, PCNA, and GAPDH antibodies. The level of Nkx2.5 was reduced to less than 5% in antisense RNA-treated cells (n = 3, *P* = 0.002). Similarly, GATA4 and MEF2C were downregulated significantly while no difference was detected in the level of PCNA. β-catenin was upregulated in the myocytes treated with AdXKN (n = 3, *P* = 0.016). (C) Western blot analysis of 20-wk old cardiac myocytes treated with increasing concentration of AdXKN showed changes in GATA4 and β-catenin that corresponded with the level of NKX2-5. Lane 1 represents cells treated with AdGFP control at 8 MOI. (D) Immunostaining of cardiomyocytes with MF20 (myosin heavy chain) revealed a reduction in the amount of myosin heavy chain (indicated by arrows) and with a punctate pattern of staining in cells exposed to antisense NKX2-5 for 48 hrs (AdXKN) compared to control cells (AdGFP).

We further studied the regulation of β-catenin and GATA4 by NKX2-5 in ventricular myocytes. The myocyte cultures were >90% α-MyHC positive as determined by immunocytochemistry (Fig. 2A). The myocyte cultures were treated with NKX2-5 antisense RNA produced from an adenovirus. The cells exposed to antisense RNA showed >95% reduction in NKX2-5 protein levels 48 hours post-treatment, while the level of PCNA control was unaffected (Fig. 2B). Antisense inhibition of NKX2-5 led to a significant increase in β-catenin protein level suggesting that NKX2-5 negatively regulated β-catenin, while expression of GATA4 and MEF2C was suppressed, suggesting a positive regulation by NKX2-5 (Fig. 2B). Furthermore, β-catenin and GATA4 protein level changes were dependent on the concentration of antisense NKX2-5 (AdXKN) used in the experiments (Fig. 2C). Cardiomyocytes treated with AdXKN for 48 hrs demonstrated reduction in the expression of myosin heavy chain, an indication of disruption in cardiac cell sarcomeric structure and function (Fig. 2D). No other morphological differences were detected between the AdXKN-trandsfected cells and controls after 48 hrs.

### NKX2-5 binds to the β-catenin and GATA4 promoters

**Figure 3:**
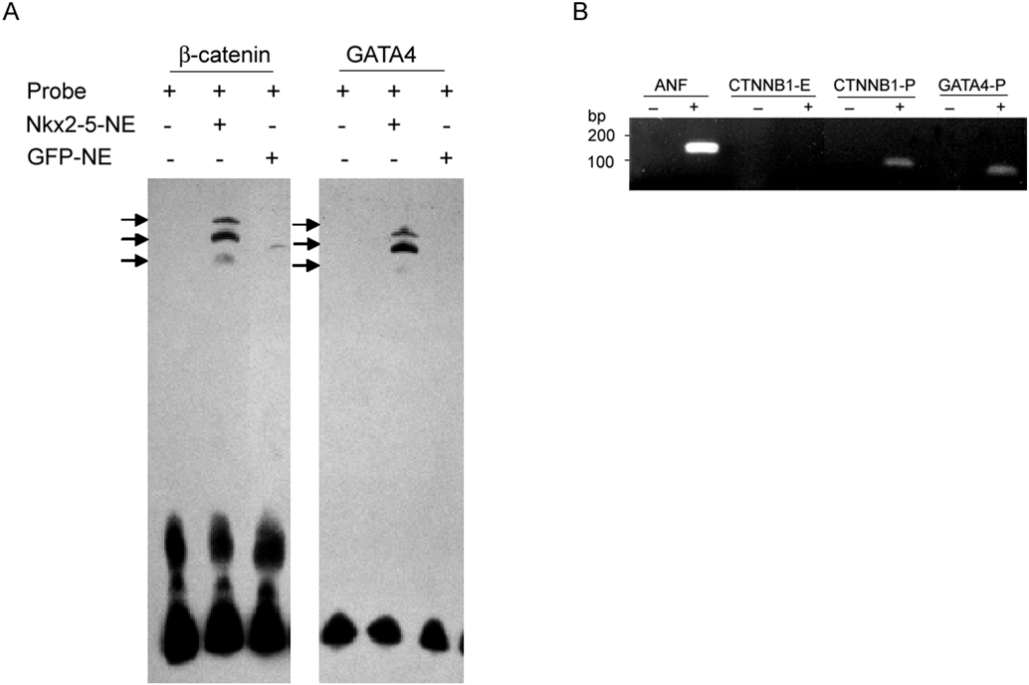
EMSA and ChIP assays indicate direct binding of NKX2-5 to β-catenin and GATA4 promoters. (A) EMSA was performed to demonstrate binding of NKX2-5 to NKE1 (within β-catenin promoter) and NKE-G (within GATA4 promoter). Arrows indicate specific bands that are detected only in nuclear extract (NE) from C2C12 cells expressing Nkx2-5. Faint band detected with C2C12+GFP nuclear extract might represent binding of endogenous Nkx2-5 to the oligonucleotides. (B) ChIP analysis using anti-Nkx2.5 antibody resulted in precipitation and amplification of DNA harbouring β−catenin (CTNNB1-P) and GATA4 (GATA4-P) promoters encompassing the NKEs. ANF promoter and β-catenin exon 4 (CTNNB1-E) sequences were used as positive and negative control respectively. Also “−” represents IgG control and “+” represents the samples where anti-NKX2-5 antibody was added, for each experiments.

To study direct binding of NKX2-5 to the *β-catenin* and *GATA4* promoters through the putative NKEs, we performed electrophoretic mobility shift assay (EMSA) using nuclear extract from retrovirally-transduced C2C12 cells overexpressing NKX2-5 [13]. The binding of NKX2-5 to NKE1 within β-catenin and to NKE-G within GATA4 promoter were examined and detected only in the presence of nuclear extract containing NKX2-5 protein (Fig. 3A). These results were further confirmed by chromatin immunoprecipitation (ChIP) on cultured cardiac myocytes (Fig. 3B). A known NKX2-5-binding site in the atrial natriuretic factor (ANF) promoter region [14], was used as positive control, and primers amplifying the 4^th^ exon of *β-catenin* (CTNNB1-E) was used as negative control in ChIP analysis. The DNA harboring the candidate NKE sequences in the promoters of *GATA4* and *β-catenin* genes were immunopreceipitated (Fig. 3B). This further confirms physical interaction of NKX2-5 to the candidate NKEs in the promoters of GATA4 and β-catenin.

### Functional characterization of NKX2-5 binding sites in GATA4 and β-catenin promoters

**Figure 4:**
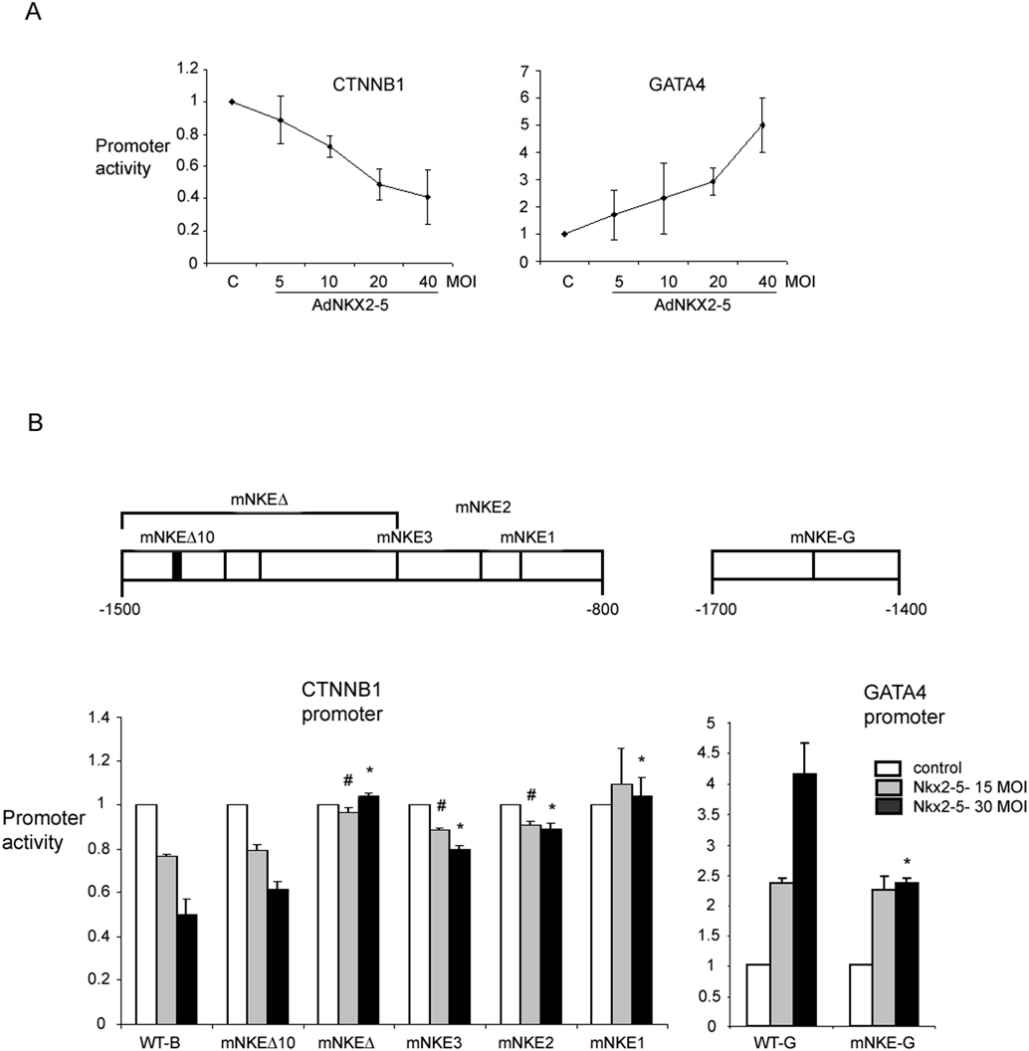
Promoter luciferase assay indicates regulation of β-catenin and GATA4 by NKX2-5. (A) The graphs demonstrate promoter activity luciferase reporter assay. For this assay, the regions between primers GF2 and GR2 for GATA4 and BF2 and BR2 for β-catenin were amplified and cloned into pGL3-promoter plasmid. Luciferase reporter assay using these constructs demonstrated repression for β-catenin sequence, and activation for GATA4 sequence, of promoter activity when the adenovirus expressing NKX2-5 was co-transfected into COS7 cells. Cells transfected with the constructs were exposed to various MOIs (5-40 MOI) of Nkx2-5-adenovirus as indicated (n = 3 for each concentration). (B) Mutational analysis of *β-catenin* and *GATA4* promoters. The approximate positions of mutated NKEs in β-catenin and GATA4 promoters and deletion of the 5′ half (mNKEΔ) of the β-catenin promoter sequence have been indicated. Luciferase reporter assay was performed using wild type (WT-B: β-catenin, and WT-G: GATA4) and mutated (mNKEs) promoters after adding adenovirus expressing NKX2-5 at either 15 (gray bars) or 30 (black bars) MOI. White bars indicate promoter activities in untreated cells. Activities of the promoters harboring mutated NKEs were all statistically different from the equally treated WT values (# and * indicate *P*<0.02) except for deletion of sequence not containing an NKE (mNKEΔ10).

In order to confirm that the identified sequences had either enhancer or repressor activity, we performed promoter-luciferase reporter assay. Regions surrounding the NKEs, the region between primers GF2 and GR2 for *GATA4*, and BF2 and BR2 for *β-catenin* indicated in figure 1, were examined for repressor or enhancer activity, in COS7 cells. The region containing NKE in *GATA4* promoter sequence led to an approximate 5 fold increase in the promoter activity, while the region surrounding NKEs in the *β-catenin* promoter sequence repressed promoter activity by 2.5 fold, in the presence of NKX2-5 expressed from an adenovirus (Fig. 4A). Similar results were obtained when mouse NKX2-5 was expressed from a plasmid in COS7 cells carrying the promoter-luciferase constructs (data not shown). To further confirm that the candidate NKEs in the *β-catenin* and *GATA4* promoters were involved in binding to Nkx2-5, we mutated or deleted the candidate NKE sequences (Fig.1 and 4B). Introduction of base changes in NKE at position −912 (mNKE1) and deletions of the region encompassing the two NKEs in the 5′ half of the sequence (mNKEΔ) in β-catenin upstream sequence completely inactivated NKX2-5 repression, while base changes at positions −948 (mNKE2) and −1065 (mNKE3) had less effect on the promoter activity (Fig. 4B). Deletion of 10 bases in the 5′ half not containing an NKE did not significantly change the promoter activity. Introducing base changes to the NKE in GATA4 promoter, reduced promoter activity by almost 50% when cells were treated with a high concentration of adenovirus expressing NKX2-5 (Fig. 4B).

### Nkx2-5 modulates Wnt/β-catenin pathway in cardiomyocytes

**Figure 5:**
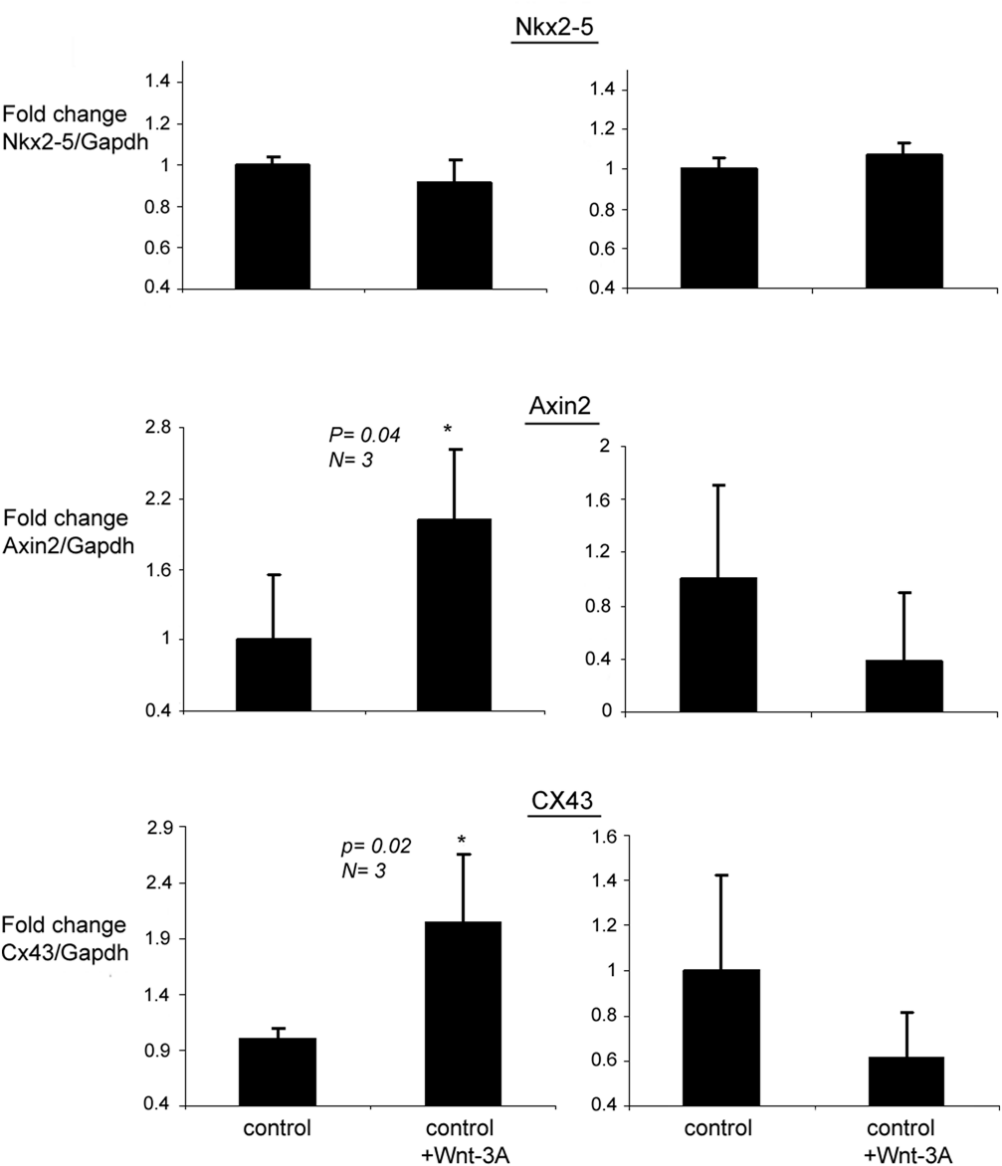
Disruption of Wnt/β-catenin pathway in mouse cardiomyocytes overexpressing Nkx2-5. Cells were transfected with adenovirus expressing mouse Nkx2-5 and concomitantly treated with mouse Wnt-3A. Treatment with Wnt-3A increased the level of Axin2 and Cx43 RNAs by approximately 2 folds, in the control cardiomyocytes. In Nkx2-5 overexpressing cardiomyocytes, upregulation of Axin2 and Cx43 by Wnt-3A was blunted.

To further study the Nkx2-5 regulation of Wnt/β-catenin pathway in cardiomyocytes, mouse Nkx2-5 was overexpressed in mouse neonatal cardiomyocytes using adenoviral system and concomitantly Wnt/β-catenin pathway was activated by treating cells with Wnt-3A. As demonstrated in figure 5, upregulation of β-catenin downstream target genes, Axin2 and Cx43 [15], [16] was completely blunted in cells overexpressing Nkx2-5, further supporting the functional role of Nkx2-5 in the regulation of β-catenin in cardiomyocytes.

### β-catenin and GATA4 transcript levels in heterozygous *Nkx2-5^+/−^* mice

**Figure 6:**
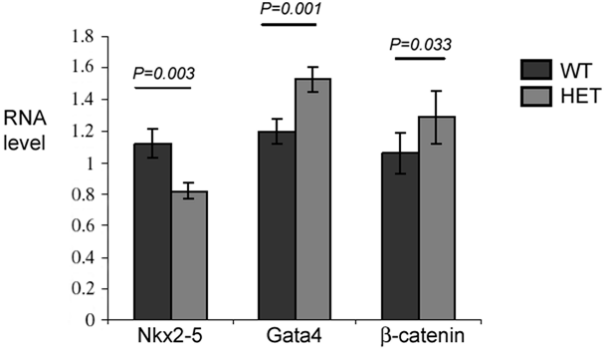
The level of Gata4 and β-catenin RNA in *Nkx2-5^+/-^* heterozygous and wild type hearts. Quantitative RT-PCR analysis of Nkx2-5, Gata4, and β-catenin in the hearts of 11.5 dpc wild type (WT) and *Nkx2-5^+/-^* (HET) embryos revealed augmentation of both Gata4 and β-catenin and reduction in Nkx2-5 RNA levels. The values (mean±SEM) for each gene were normalized against GAPDH. N = 9 for WT and N = 11 for HET hearts.

To further study the proposed regulation of GATA4 and β-catenin by Nkx2-5 *in vivo*, we examined, by quantitative RT-PCR, the level of *β-catenin and GATA4* RNAs in *Nkx2-5^+/−^* mouse embryo hearts at 11.5 dpc. Nkx2-5 RNA level in heterozygotes (*Nkx2-5^+/−^*) are decreased to approximately 80% of that in the wild type mice. An approximately 20% increase in *β-catenin* transcript level was detected. Similarly, *Gata4* RNA level was also increased (Fig. 6).

## Discussion

Nkx2-5 transcription factor regulates multiple aspects of cardiac cell structure, function, and development [5], [17], [18]. Identification of genes downstream of Nkx2-5 is therefore crucial in understanding the transcriptional network that regulates cardiac myogenesis. Following identification of candidate NKEs in the promoter of *GATA4* and *β-catenin* genes; using EMSA and ChIP, we demonstrated that Nkx2-5 binds to the region surrounding identified sequences. Furthermore, gene reporter assay revealed that the regions encompassing NKEs were functional and that base change or deletion of the NKEs resulted in either reduction or complete loss of activation or repression of the promoter activity when Nkx2-5 was co-expressed in COS7 cells. The data presented here indicates that NKX2-5 negatively regulates β-catenin in cardiac myocytes. β-catenin activation is the hallmark of canonical Wnt pathway (reviewed in [2]). Both Wnts and Wnt inhibitors are expressed in the developing heart suggesting a requirement for regulated Wnt signaling. At early stages of development in mouse embryos β-catenin directly regulates Islet1 expression [10], a gene essential for the development of cardiac cells in the secondary heart field, and therefore, β-catenin is essential for development of cardiac cells that originate from the secondary heart field [19]. In mouse embryonic stem cells Wnt/β-catenin appears to have a biphasic role as it enhances cardiogenesis at early stages of differentiation while conversely inhibiting cardiogenesis at later stages of development [20]. The inhibitory function of Wnt/β-catenin in cardiogenesis is supported by several lines of evidence such as formation of multiple hearts in mice in which β-catenin is ablated in the endoderm [9], and the blockage of cardiogenesis when Wnt-3A or Wnt-8C are ectopically expressed in the precardiac mesoderm of chicken and xenopus embryos [8]. Our results add to these findings and suggest that β-catenin expression is repressed in cardiac myocytes at least at the gestational ages tested and that this transcriptional repression is mediated by Nkx2-5. Previous studies have suggested the presence of a transcriptional repressor domain at the carboxyl-terminus of Nkx2-5 [11], [17] that may mediate the repressor activity of Nkx2-5 on β-catenin gene expression shown in this study. Repression of genes such as connexins 40 and 43 in murine cardiomyocytes overexpressing Nkx2-5 has been shown in other studies but the molecular nature and the possible co-factors involved are not identified. Transcriptional repression has also been demonstrated for other cardiac-associated factors such as GATA4/FOG2 complex directly repressing Lhx9 gene in the heart [21]. *In vivo*, examination of β-catenin RNA level in Nkx2-5 heterozygous hearts (reduced Nkx2-5) revealed an increase in β-catenin RNA which was in agreement with our *in vitro* data. A recent study has shown that activation of Wnt/β-catenin downregulates Hdac1, a suppressor of Nkx2-5 and GATA4 in cardiomyocytes [22]. Therefore, in Nkx2-5 heterozygous hearts, higher levels of β-catenin could increase Nkx2-5 and GATA4 via downregulation of Hdac1. It is note worthy that in Nkx2-5 heterozygous hearts the level of Nkx2-5 is not exactly half as compared to the wild type hearts. Further study is required to delineate the regulation of β-catenin in cardiac myocytes originated from primary (e.g. left ventricle) or secondary (e.g. right ventricle) heart fields and at different developmental stages.

Moreover, our study indicated the direct regulation of GATA4 by NKX2-5 in human fetal cardiac myocytes. The role of GATA4 in early cardiogenesis is clearly established [3], [5], [23]. GATA4 inactivation results in myocardial thinning and disruption of myocyte cell proliferation [24]. The presence of high affinity GATA4 binding sites in cardiac enhancer regions of the *Nkx2-5* promoter [25], [26] have suggested that *Gata4* is genetically upstream of *Nkx2-5*. Accordingly, the expression of GATA4 is upregulated prior to Nkx2-5 in P19CL6 cardiomyocyte model [27].Treatment of *in vitro* cultured ventricular myocytes with antisense NKX2-5 in this study, however, led to a reduction in GATA4 level suggesting that NKX2-5 positively regulates the expression of GATA4, at least in cultured cardiac myocytes, and therefore, not only does GATA4 regulate NKX2-5, but the converse regulation exists as well. GATA4 RNA level in Nkx2-5^+/−^ embryos, however, was unexpectedly upregulated. We reason that this upregulation represents a complex regulation of GATA4 transcription factor during development. Since GATA4 begins to express in cardiac progenitor cells prior to Nkx2-5 [27], its expression may be controlled by unknown factors that are upregulated in the Nkx2-5 heterozygous hearts due to a compensatory mechanism, and therefore, higher expression of GATA4 in the Nkx2-5 heterozygous hearts may be essential to maintain Nkx2-5 above a threshold level necessary for the normal development of the heart.

**Figure 7:**
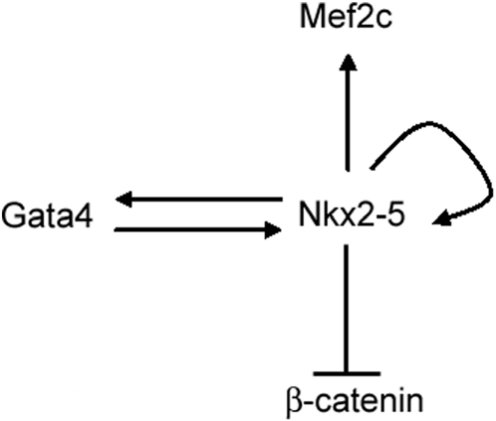
A proposed model demonstrating the regulation of β-catenin, GATA4, and MEF2C by Nkx2-5. Regulation of Nkx2-5 by GATA4, and its autoregulation has been shown in previous studies.

Overall, our study proposes a model where Nkx2-5 negatively regulates β-catenin and positively regulates GATA4 in cardiomyocytes, as depicted in figure 7. The *in vitro* cell culture model of fetal cardiac myocytes, such as the one used in this study, in combination with antisense RNA or siRNA technology [28] helps to elucidate the transcriptional regulatory network in cardiac myogenesis.

## Materials and Methods

### Cardiac myocyte culture

Human fetal hearts at gestational ages 20–22 (n = 5) weeks were collected in IMDM+20% FBS. Ventricles were separated and the fat and connective tissues were removed before mincing. The tissue was then digested by incubating in enzyme solution (1 mg/ml collagenase type II, 0.125% trypsin, 0.03% glucose) for 10 min at 37°C and passed through a filter. These steps were repeated 2–3 times and the cells were pelleted by centrifugation. The cell mixture was resuspended in the media and the number of live cells was counted. Fibroblasts were separated from other cardiac cells using anti-fibroblast antibody-conjugated magnetic beads purchased from Miltenyi Biotech. Inc., according to manufacturer's instruction. Cells were cultured in IMDM+20% FBS for 3–5 days at 37°C and 5% CO_2_^.^

Mouse neonatal cardiomyocytes were prepared using similar protocol. Isolated cells obtained after tissue digestion were pre-plated for 1 hr to allow attachment of fibroblasts to the plate. Cells not adhered were then seeded on plates coated with 0.1% gelatin and cultured in DMEM+15% FBS for 24–48 hrs before they were used in experiments.

### Ethics Statement

Fetal cardiac cells were derived from terminated pregnancies under an approved protocol by the Hospital for Sick Children Research Ethics Board. Written consent was obtained from mothers who participated in the study. The use of mice in this study was also approved by an institutional ethics board.

### Immunofluorescence and western blot analyses

Immunofluorescence and western blot analyses were performed according to the standard procedures. Myosin heavy chain antibody (MF-20) was obtained from the Developmental Studies Hybridoma Bank developed under the auspices of the NICHD and maintained by the University of Iowa, Department of Biological Sciences, Iowa City, IA 52242. Other antibodies were purchased: PCNA (DAKO), GAPDH (Novus Biologicals), β-catenin (Upstate Biotech.) and Nkx2-5, GATA4, and MEF2C (Santa Cruz Biotechnology). For western blot analysis one to three plates of cardiac myocytes, for each experimental condition, were lysed with RIPA buffer and the samples were separately loaded on SDS-PAGE gel and blotted.

### Production of sense and antisense NKX2-5 adenovirus and treatment of myocyte cultures

Human NKX2-5 cDNA was amplified from fetal heart RNA by RT-PCR using one-step RT-PCR kit (Qiagen) and using primers: agactggtcgactgccaccatgttcc and agagtcagggatcctagttgaggtg, and digested with *Sal*I/*Bam*HI. NKX2-5 cDNA was cloned into *Sal*I/*Bgl*II site of pAdTrack-CMV (Stratagene Inc.) in forward and reverse orientations. Manufacturer's instructions were followed to produce the virus. The virus titer and multiplicity of infection (MOI) were estimated using HEK-293 cells and by calculating the number of GFP^+^ cells. The recombinant adenoviruses were used at different MOIs.

Adenoviral vector to express mouse Nkx2-5 was constructed by amplifying mouse Nkx2-5 cDNA using primers: aacctgcgtcgaccaccatgttcc and cggagaaaggatcccaggcctgcg and cloning the cDNA into NotI/SalI sites of pAdTrack-CMV.

### Computational analyses for 5′-flanking sequences

TFSEARCH (http://www.cbrc.jp/research/db/TFSEARCH.html) was used to find putative NKX2-5 binding elements in 2kb upstream of the transcriptional initiation sites of human *GATA4* (accession no. AC090790) and *β-catenin* genes (accession no. AY463360.1).

rVISTA (regulatory VISTA, http://genome.lbl.gov/vista/index.shtml) was used to examine the similarity between human and mouse sequence in the 5′-flanking region of each gene.

### Wnt-3A treatment of cardiomyocytes and expression of Axin2 and Connexin 43

Mouse neonatal cardiomyocytes were prepared as described above. Cells were transfected with adenovirus expressing mouse Nkx2-5 at 1 MOI and cultured in growth media for 24 hrs. Cells were then treated with mouse Wnt-3A (R&D Systems), at a concentration of 100 ng/mL for 16 hrs in media containing 1% serum. RNA was isolated using Trizol (Invitrogen) and cDNA was synthesized. Gene expression was assayed using either pre-designed Taqman assays or oligonucleotides with SYBR Green. The following sets of primers were used: Nkx2-5 (Mm00657783_m1, Applied Biosystems), Gapdh (Mm9999915_m1, Applied Biosystems) Axin2 (tggaggaaaatgcctaccag, acatagccggaacctacgtg), Cx43 (gaacacggcaaggtgaagatg, gagcgagagacaccaaggaca), and Gapdh (tccaccaccctgttgctgtag, gaccacagtccatgccatcact). Expression levels determined in each assay were normalized against Gapdh. Data were analyzed by relative quantitation method using standard curve.

### Electrophoretic mobility shift assay (EMSA)

EMSA was performed using Lightshift Chemiluminescent EMSA kit (Pierce). Nuclear extracts were prepared from C2C12 myoblasts expressing either human Nkx2-5 or GFP [13] using method previously described [29]. Complementary oligonucleotides encompassing the putative NKX2-5 binding sites in β-catenin (NKE1: tataagaattaacctgcagacagcgctctg) and GATA4 (NKE-G: agaagaaaccctaagtgtgtcgcccccagc) promoters were synthesized and annealed. The annealed oligonucleotides were biotinylated using 3′ end DNA labeling kit (Pierce) and incubated with 5 µg of nuclear extracts for 20 min to allow binding. The mixtures were analyzed by gel electrophoresis on 5% PAGE in 0.5× TBE buffer. The membrane was hybridized with HRP-conjugated straptavidin (Pierce) for detection of DNA-protein complex according to the manufacturer's instruction.

### Chromatin immunoprecipitation (ChIP)

ChIP was performed according to a published protocol [30] with some modifications. In brief, cultures of cardiac myocytes were fixed using formaldehyde. The cells were lysed with RIPA buffer and sonicated to fragment DNA. DNA-protein complexes were immunoprecipitated with 1 µg of Nkx2-5 antibody (Santa Cruz) and protein G (Roche), according to the manufacturer's instruction. The immunoprecipitated DNA was used as template in PCR using primers: ANF: (agtaagaatgcggctcttgc), (gagacagaaccctccccatt), CTNNB1-P: (tcgacaaacgtcaattttgc), (tcgattaagcagcctccaat), GATA4-P: (cccttcagccttaagttcc), (gcgacacacttagggtttcttc), and CTNNB1-E (cacccggaggtgatttacaca), (gcctggctctgatctccatgt). Amplification condition was 94°C- 4 min, 94°C- 30 sec, 50°C-30 sec, 72°C- 30 sec, for 35 cycles.

### Transfection and reporter gene assays

Promoter regions containing the candidate NKX2-5-binding sites in β-catenin (CTNNB1) and GATA4 promoters were amplified using primers GF2 (gagtccgaagagctcgcagattgg), GR2 (ggcgcaaactcgaggaaaggaaac), and BF2 (ggcagttgagctcttaccacttata), BR2 (ggctgtgaactctcgagtagaacg) and cloned into the *Sac*I/*Xho*I sites of pGL3-promoter plasmid (Promega). Plasmids were sequenced to confirm the correct DNA sequence. COS7 cells were grown in DMEM+10%FBS and co-transfected with pCMV-lacZ plasmid and either pGL3-GATA4 or pGL3-βcatenin constructs using FuGENE 6 (Roche Applied Science).

Mutation or deletion of Nkx2-5-binding elements was carried out by amplifying the promoter from the wild type reporter construct using oligonucleotides harboring modified DNA sequences (containing *Pst*I sites) and primers on both sides of the promoter sequence and surrounding the cloning site of pGL3 vector: pGL3-1 (ctagcaaaataggctgtcccag) or pGL3-2 (ccaagcttacttagatcgcaga). The amplified DNA fragments were then digested with either *Sac*I/*Pst*I or *Pst*I/*Xho*I and subcloned into the *Sac*I/*Xho*I sites of the pGL3-promoter vector. Oligonucleotides used to amplify mutated constructs: mNKE1: aattggaggctgctgcagatagctttctcta, mNKE2: ttgatacctagtgactgcaggaaccagataa, mNKE3: ctataagaattaactgcagacagcgctctgg, and their complementary sequences. To make mNKEΔ, the mNKE3 construct was cut with *Pst*I and *Sac*I and blunted with Klenow DNA polymerase. The DNA fragment encompassing the promoter sequence downstream of mNKE3 site was eluted from gel and cloned into the *Sma*I site of pGL3-promoter.

Two to five micrograms of wild type or mutated constructs were used for transfecting COS7 cells. Cells were then exposed to different MOIs of adenovirus expressing NKX2-5, one hour after the start of the transfection. The treated cells were lysed with Promega Reporter Lysis Buffer and luciferase activity was measured using a luminometer. The β-galactosidase activity was measured and used for normalization of the values.

### Analysis of *Nkx2-5^+/−^* embryos

Animal procedures were performed under approved institutional protocol. Heterozygote *Nkx2-5* embryos were generated by breeding *Nkx2-5^+/−^* heterozygote mice. Embryos were genotyped as previously described [31]. RNA was prepared from hearts of 11.5 dpc *Nkx2-5^+/−^* embryos and used in TaqMan PCR assays (Applied Biosystems) to detect levels of Nkx2-5 (Mm00657783_m1, Applied Biosystems), Gata4 (Mm00484689_m1, Applied Biosystems), β-catenin (Mm00483033_m1, Applied Biosystems), and Gapdh (Mm9999915_g1, Applied Biosystems) RNAs. Data were analyzed by relative quantitation method using standard curve.

### Statistical analysis

Results are expressed as mean±SD unless otherwise indicated. Statistical significance was determined by one-way ANOVA. *P*<0.05 was used to indicate statistical significance.
